# A pattern language of compassion in intensive care and palliative care contexts

**DOI:** 10.1186/s12904-019-0402-0

**Published:** 2019-02-02

**Authors:** A. L. Roze des Ordons, L. MacIsaac, J. Everson, J. Hui, R. H. Ellaway

**Affiliations:** 1 Department of Critical Care Medicine; Division of Palliative Medicine, Department of Oncology; Department of Anesthesiology, Cumming School of Medicine, University of Calgary, South Health Campus ICU, 4448 Front St SE, Calgary, AB T3M 1M4 Canada; 2 Alberta Health Services, Palliative Care Consult Service, Calgary Zone, Alberta Canada; 3 Department of Family Medicine; Division of Palliative Medicine, Department of Oncology, Cumming School of Medicine, University of Calgary, Calgary, Alberta Canada; 4 Department of Community Health Sciences, Office of Health and Medical Education Scholarship, Cumming School of Medicine, University of Calgary, Calgary, Alberta Canada

**Keywords:** Compassion, Intensive care unit, Palliative care, Constructivist grounded theory, Pattern language

## Abstract

**Background:**

Compassion has been identified as important for therapeutic relationships in clinical medicine however there have been few empirical studies looking at how compassion is expressed different contexts. The purpose of this study was to explore how context impacts perceptions and expressions of compassion in the intensive care unit and in palliative care.

**Methods:**

This was an inductive qualitative study that employed sensitizing concepts from activity theory, realist inquiry, phenomenology and autoethnography. Clinicians working in intensive care units and palliative care services wrote guided field notes on their observations and experiences of how suffering and compassion were expressed in these settings. Data were analyzed using constructivist grounded theory.

**Results:**

Fifty-eight field notes were generated, along with transcripts from three focus groups. Clinicians conceptualized, observed, and expressed compassion in different ways within different contexts. Patterns of compassion identified were relational, dispositional, activity-focused, and situational. A pattern language of compassion in healthcare was developed based on these findings.

**Conclusions:**

Recognizing compassion as shifting patterns of diverse attitudes, behaviours, and relationships raises numerous questions as to how compassion can be developed, supported and recognized in different clinical settings.

**Electronic supplementary material:**

The online version of this article (10.1186/s12904-019-0402-0) contains supplementary material, which is available to authorized users.

## Key points for decision-makers

- The context of clinical practice influences how compassion is expressed.
- Compassion can be enacted in different ways; in relation with others, in one’s disposition towards others, in specific clinical activities, and in response to particular clinical and social situations.
- Reflecting on expressions of compassion can help clinicians become more mindful of compassion and its deficits within the clinical setting.

## Background

Compassion is generally seen as a desirable yet complex social phenomenon with many forms and dimensions. Sinclair et al. described compassion as a virtuous response that seeks to address suffering and facilitate healing through relational understanding and action [1]. Compassion has also been associated with caring, shared understanding or empathy, and a way of alleviating both emotional and physical distress [2, 3]. Despite the absence of a universal definition, compassion is understood as involving recognition and mitigation of suffering [4]. Greater compassion has been associated with increased work satisfaction [5] and improved patient outcomes [6, 7].

Conversations about compassion in healthcare have mainly focused on where it is lacking or how it can be developed and nurtured [8, 9]. Measuring or accounting for compassion (or its absence) in a systematic and objective way is an elusive challenge, not least because of the reciprocal and relational nature of compassion. How compassion is understood also varies with clinical context. For instance, compassion is central to palliative care, and in recent years these approaches have been infused into end of life care within the intensive care unit (ICU) [10]. Compassion need not be restricted to end of life care; all patients could benefit from relationship-centered approaches that integrate medical treatments with individualized holistic support [11]. While patients, families, and clinicians value compassion, its inconsistency in acute care settings has been lamented in both popular media [12, 13] and the medical literature [14–17]. Challenges to compassionate care in the ICU include a biomedical focus; gaps in interprofessional communication; workplace-related conflict; discord between patients, families, and clinicians; resource constraints; and personal life stresses [18].

Although many perspectives on what compassion is have been described [1–5], few studies have looked at how compassion is expressed in this context or how context shapes its expression. Nevertheless, clinician perceptions and beliefs about compassion likely vary within differing contexts as compassion is intrinsically bound to the physical, social and cultural milieu [19, 20].

Previous studies have considered how context modulates compassion [19, 20]. This study was undertaken to better understand how clinicians in ICU and palliative care settings perceive expressions of compassion.

## Methods

### Methodology

In approaching this study we were challenged by being interested in both experience and practice, and the need for a highly inductive approach in exploring these phenomena. To that end, we designed the study around inductive inquiry [21] that drew on procedures from constructivist grounded theory that are based on guiding “interpretive theoretical practice” [22]. We also drew on a number of sensitizing concepts to “draw attention to important features of social interaction and provide guidelines for research in specific settings.” [23] These included using activity theory to reflect on the mediating and social nature of practice, [24] realist inquiry to focus on the role of context in shaping compassion, [25] phenomenology to consider the perceptual and experiential aspects of compassion in the workplace, [26] and autoethnography to consider how compassion was enacted [27]. These sensitizing concepts afforded the ontological breadth we needed to encompass the phenomena of interest while constructivist grounded theory methods weaved them together to provide epistemological coherence. It is important to note that we were not specifically seeking to build theory in designing the study, nor were we following one of the many interpretations of grounded theory principles [28]; rather, we sought to capture the breadth of issues associated with the ways in which clinicians perceive compassion in their everyday working lives.

### Study setting

The study was conducted at five clinical sites (including 4 ICUs and 3 palliative care services) in Calgary in Western Canada. All ICUs were located in academic teaching hospitals. One of the palliative care services was an inpatient palliative care unit and the other 2 were consult services. Five clinicians who worked in these settings episodically observed and recorded their reflections on instances of suffering and the associated responses. The study was conducted between March 2016 and January 2017.

### Data collection

We developed a field notes (FN) template based on the intersecting sensitizing concepts outlined above. We iteratively tested and piloted the template for clarity and breadth (Additional file 1). The template was used to collect structured field notes and reflective commentaries, which were anonymized and de-identified prior to analysis.

### Participants

We purposively recruited participants who had an interest in critically reflecting upon compassion, and to ensure diversity in discipline and practice contexts. There were five participants: one nurse clinician, one nurse practitioner, and three physicians. Informed consent was obtained from all individual participants. Data collection was informed by participatory methods, in which several members of the research team were also subjects of the research; cultural ethnographic methods of observation; elements of constructivist grounded theory, where observations and interpretations arose from interactions between participants as researchers within their usual workplace settings; and principles of phenomenology, to enable understanding of individual experiences. Participants were asked to observe and reflect upon instances of suffering and compassion that captured their attention as they engaged in routine clinical work, attending to their own interactions as well as those in which they were not directly involved; they were not provided with specific instruction on how to choose which instances to record. Observations and reflections were recorded discretely in brief during patient care and retrospectively in greater detail when outside of the clinical setting, guided by the field notes template. Data collection and analysis occurred concurrently, and evolving interpretations and concepts from the field notes were explored with the participants in a focus group (FG, 3 participants) or individual interview (IN, 2 participants); participant experiences as observers and the impact of observing and reflecting on their understanding of these phenomena were also explored (Additional file 2). The focus group and interviews were audio recorded and transcribed.

### Data analysis

Two members of the research team (ALR, RHE) applied constructivist grounded theory methods to analyze the field notes and interview transcripts [22, 29]. Analysis began by reading through the transcripts and listening to focus group and interview recordings to become familiar with the data. Data was then coded line by line using inductive and iterative coding techniques, with constant comparative analysis. The analysis team met after analyzing every third field note and after each interview to negotiate codes and develop a code book; ambiguities were resolved through open discussion. After initial coding had been completed, the researchers performed axial coding by exploring relationships between codes; these codes were then organized into themes. Emerging theories were developed through iterative group writing and discussion. In considering codes, organizing our data into themes, and developing emerging theories, we used concepts from activity theory to consider how collaborative activities in healthcare are mediated and realist inquiry to explore how context shapes perceptions of compassion. We drew on the patterns of clinical context model [30] to capture the breadth of contextual factors that may influence perceptions of compassion. We also used concepts from phenomenology to consider clinicians’ perceptions and autoethnography to look at participants’ descriptions of their own shared practice and longitudinal involvement in the research process.

## Results

The five investigator-observers generated 58 reflective field notes (Table 1). Compassion was conceptualized, observed, or expressed in different ways, depending on context and the observer’s perspective. Four patterns of compassion were identified: relational, dispositional, activity-focused, and situational. We have indicated below the participant associated with each quote and the context of their remarks (field note (FN), focus group (FG), or interview (IN).

**Table 1:** Participant backgrounds and contributions

Participant^a^	Role	Number of reflective field notes								
		Total	Palliative Care				ICU			
			A	B	C	D	a	b	c	d
P1	Physician, critical care	20	0	0	0	0	0	9	0	11
P2	Physician, critical care	1	0	0	0	0	0	0	1	0
P3	Nurse practitioner, critical care	4	0	0	0	0	4	0	0	0
P4	Physician, palliative care	13	1	0	1	11	0	0	0	0
P5	Physician, palliative care	10	9	1	0	0	0	0	0	0
P6	Nurse clinician, palliative care	10	0	0	0	10	0	0	0	0
Total		58	10	1	1	21	26	9	1	11

^a^One of the participants works in both ICU and palliative care settings and was assigned a different participant number for each setting to preserve anonymity. ICU – intensive care unit. A – Palliative care unit, 1500 bed hospital; B – Palliative care consult service, 1500 bed hospital; C – Palliative care consult service, 650 bed hospital; D – Palliative care consult service, 269 bed hospital; a – ICU, 28 bed unit; b – ICU, 18 bed unit; c – ICU, 10 bed unit; d – ICU, 10 bed unit

### Relational

While patients provided much of the context for compassion, acts and experiences of compassion were not only directed towards patients, but also between members of the healthcare team through debriefing and mentorship. For instance, one physician described words of wisdom for a learner who was struggling with the balance between developing a therapeutic relationship with his patients while not becoming paralyzed by sorrow: *“It is in caring about people that we can be of benefit as physicians, that in doing so we learn from them, and in being human we can’t help but be affected by them, that we need to find ways to accompany them without taking on their burden.”* (P1, FN).

Compassion was also described in terms of connections with others: *“Compassion, for me, seems to be embedded within relationship, within conversation, and within that sort of therapeutic listening and presence.”* (P6, FG) This sense of connectedness was thought to have the capacity to transcend roles: *“Compassion wasn’t necessarily separated, you know, by scopes of practice or a professional designation. I kind of sensed that it was within the limits of human connectedness.”* (P6, FG) Connectedness could be established *“on whatever level is possible.”* (P4, FN) A kind and caring attitude was considered as a foundational element of compassion: *“You endeavour to show them that you care, show them that you will do your best.”* (P3, IN) Connectedness was expressed both verbally through choice of words, and non-verbally through how words were spoken, positioning, and touch: *“The patient’s wife was standing by his bed and looking worried; the NP said to her, “It’s hard to see him like this.” His wife nodded and became tearful and the NP gave her a hug.”* (P5, FN).

Participants noted the integrative nature of compassion in clinical care: *“Compassion is the most integrative and it’s the hardest piece to put together.”* (P3, IN) Integration was further described as taking a holistic approach: *“Being able to look at the whole picture as opposed to sort of what’s right in front of you or what you’re needing to address as a healthcare provider in that moment… being able to understand the context and the whole story of that person.”* (P6, FG) Integrative compassion was also facilitated through self-awareness and reflection: *“You’re reflecting on what’s going on inside, but also reflecting on what would compassion mean to this patient, or this person. What would be helpful? What is the suffering?”* (P6, FG).

### Dispositional

In some instances, compassion was described as a feeling that emerges between people: *“I believe there are a lot of compassionate acts that people aren’t overtly doing, it could be just something the person needs time and needing the room to notice, right? Or not obvious to anyone. So, there’s really subtle things that sometimes - I think compassion can just be feeling.”* (P4, FG) Another example described the *“shared burden of suffering”* (P3, FN) experienced in talking with a patient. Compassion was also described as a way of being, beyond action. *“Whatever your scope of professionally defined practices, it’s … how you are as opposed to what you’re doing.”* (P5, FG).

Compassion was seen by some as requiring honesty. This was reflected in conversations with patients and their families regarding prognoses and the associated uncertainty: “*I talked with him, saying I wanted to be honest about what I was thinking, that he was becoming sicker and I worried he may not make it home.”* (P5, FN) Honesty was also conveyed by clinicians expressing their own vulnerability: *“A family medicine resident working with me visited [patient] every day, and I sensed that a connection had formed between them. I had not truly realized the impact of this on the resident until the last day of the rotation when we sat down to talk about how the rotation had gone, and he began to cry … he was saddened by witnessing the suffering that people endured.”* (P5, FN).

Participants described acceptance of others’ perspectives and experiences without judging or attempting to placate suffering or the sadness of loss: *“I acknowledged the void that would be felt in his family with a prolonged hospital stay and foreshortened future.”* (P3, FN) Similarly: *“I’ll sit with them. I’ll acknowledge their pain, their loss, their anxiety, their hope, their destitution.”* (P2, FG).

### Activities

Participants commented on how compassion was expressed through action: *“Whether or not it’s in your head, you have to enact it … it’s not enough to feel compassionate.”* (P4, FG) In some instances, compassion was shown by going above and beyond what was expected, while in other situations, compassion was conveyed through small acts of kindness: *“I recognized how simple gestures such as leaving a note is respectful and lets the person know he’s being thought of, that someone cares enough to want to come back.”* (P4, FN).

Compassion was also conceptualized as a skill that can be developed: *“Observing this confirmed my belief that people are very capable of compassion and responding to suffering.”* (P4, FN) As a skill, participants felt compassion could be applied deliberately. *“There are deliberate acts of compassion. Outside of this global niceness. It can be a skill as well.”* (P4, FG) Considering compassion as a skill may facilitate teaching, yet introduces an ethical dimension, as described by the following passage:

*“So if someone acts compassionately … yes, you’re compassionate, although maybe they’re not actually feeling compassionate. Or maybe the other person, the patient, doesn’t feel any sense of compassion at all, but still they’re doing all these things that if you look at a checklist you would say, yes, they’re compassionate. I think it becomes difficult when you consider it as a skill and maybe there are some skill elements to it … maybe that’s what you can make explicit to trainees.”* (P5, FG)

Compassion was further conceptualized as an art. For instance, one participant remarked that compassion cannot simply be learned as an abstraction – observing and learning from role models who skillfully relate to others in a sensitive and caring way is essential: *“I think I was taught the theory behind it. I don’t think you can teach compassion from a textbook; I think it’s probably a more tacit knowledge that you learn from the artists and you shape the way.”* (P3, IN) Another passage commented on integrating compassion into medical emergencies, where the focus on rescuing may overlook the patient’s experience: *“I still think there’s room to be compassionate and cognizant of where people are without sacrificing [clinical care]. I think the artists of our group do it beautifully; we’re not all artists.”* (P3, IN).

### Situational

Compassion was described as emerging through interactions within a given situation: *“It is emergent within the moment… something you need to attend to and yet something you can’t necessarily pre-empt. There are different ways of responding. You have to be mindful and present in responding to the needs in the moment.”* (P5, FG) Similarly: *“You find out where the needs take you.”* (P2, FG) Participants became more aware of this emergent nature of compassion over time: *“there’s that flow and ebb between patients and families and health care providers and I think that was the one thing that as an observer you became more attuned to or aware of.”* (P6, FG) The emergent nature of compassion required a degree of authenticity: *“It’s in the moment, and I think for it to be perceived as compassion it needs to be authentic.”* (P6, FG).

Compassion was also identified as being contextual, modulated by others’ responses, the relationship, the circumstance, and the subtleties of the environment, thereby limiting the utility of planned or standardized approaches: *“To have a compassionate approach isn’t a recipe card, right? It’s very context driven.”* (P6, FG) Participants noted that context can introduce complexity in expressing compassion.

Compassion was seen by some as requiring presence. This was reflected in caregivers showing interest in patients and families, colleagues, and learners as people, honoring their life stories and values: “*Compassion is really embedded in … just being present to people. Being present to their suffering. It’s embedded in just the way we are with them … what is important and meaningful and purposeful in their lives. What their experiences have been.”* (P6, FG) Recursive presence over time was another way in which compassion was expressed: *“Having the attending hospitalist spend time with the family and explain what was happening earlier in the morning and then to check in with them later made them feel valued and supported.”* (P6, FN) Similarly: *“I said I would come check on him in hospital again and that I looked forward to hearing about how his day passes were going. He grinned and held his hand out to shake on it.”* (P1, FN) Presence was also bound to the temporal nature of care: “*I had a sense she wanted to keep talking, realizing that when she stopped, the meeting would come to a close and life support be discontinued, resulting in her husband’s death.”* (P1, FN).

**Fig. 1:**
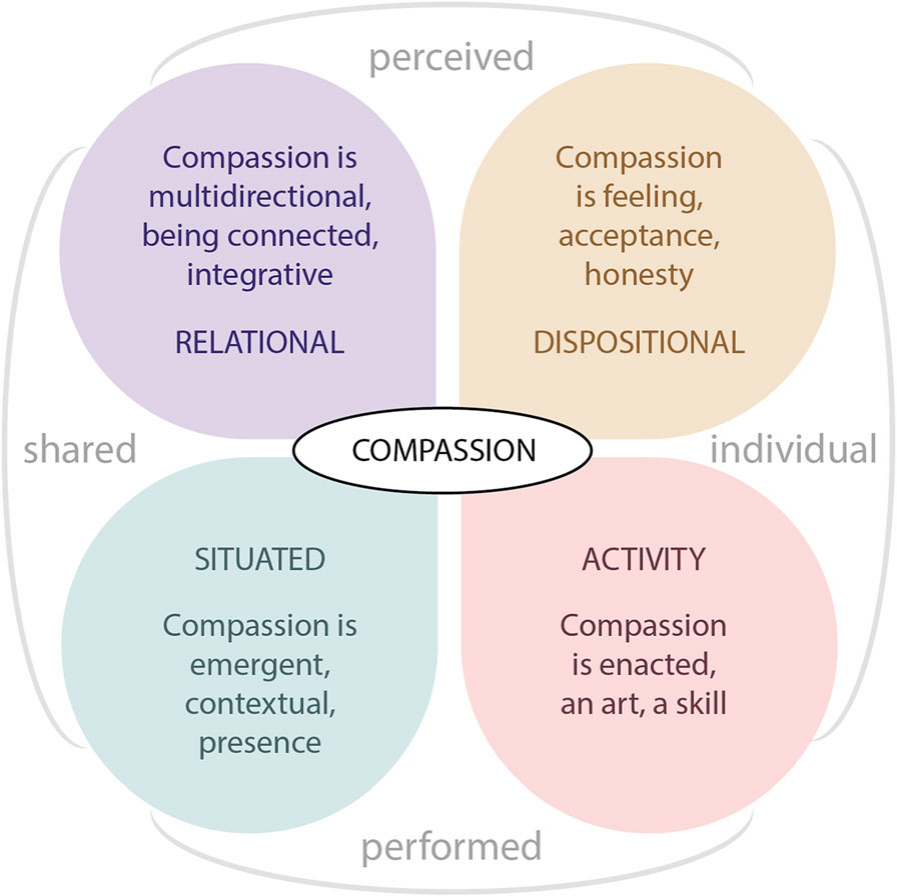
Dimensions of compassion identified in this study. We identified multiple facets of compassion that can be represented using two orthogonal axes; perceived-performed and individual-shared

A diagrammatic representation of the dimensions of compassion and their relationships to each other is depicted in Fig. 1. From this, we can understand our themes as facets of compassion. While any given instance of compassion may reflect different combinations of these facets, ‘pattern language’ [29, 30] can be used to describe different instances of compassion. Patterns are regularities perceived in the world that can be used describe the relationship between a problem, the context, and a solution [30]. A ‘pattern language’ represents a group of patterns that can be combined and connected in ways that collectively describe different expressions and dimensions of a given phenomenon, and that can, in new combinations, help to generate new and innovative ways of seeing and working with the phenomenon [30]. We return to this point in the discussion.

One additional factor we identified that did not fit the dimensional model above was the tension between compassion being both an essential part of practice and an intrinsically modified approach to practice. This tension was realized across many of the facets we identified.

Compassion was often seen as a non-negotiable essential part of being a clinician: “*It’s a daily requirement. It’s a daily need … without compassion, medicine is very mechanical.”* (P2, FG) To that end, compassion was described as central to a person’s experience, yet also underappreciated: *“For the experience of the individual, compassion is essential … it’s not something you’re aware of all of the time, and it's not until you become aware of its absence that you realize the importance of it.”* (P5, FG) Participants noted that some of the more subtle acts of compassion were not always noticed immediately within the clinical setting, often identified as such only in retrospect.

We also found a contradictory sense that compassion is enacted or perceived as being above and beyond ‘usual’ practice. This was partly related to compassion being more apparent in the moment when there were substantial modifications to what were considered routine behaviours and attitudes; modifications were behavioural, dispositional and relational.

Many of the modifications were *behavioural*, such as pausing clinical activities to acknowledge or address patient concerns or needs, discussing options with the patient or their family members, or teaching others about the emerging situation. At other times modifications involved adapting medical care in response to dynamic circumstances or new information, such as transitioning from invasive lifesaving measures to a palliative approach to care when the likelihood of recovery became too remote. Sometimes compassion was reflected in avoiding actions whose impact could inflict suffering and cause more harm than benefit:

*“He had not been allowed to eat or drink for the past 3 days as the medical team was worried about aspiration and wanted a swallowing study first. I told him he was at risk of food going into his lungs, although he could eat whatever he wanted to for comfort, even pizza, as our goal was shifting to focus on quality of life and making the most of the time he had left.”* (P1, FN)

At other times compassion was reflected by reassuring patients, families and colleagues:

“*The physician told me she was very worried the patient would not survive, berated herself for not pushing the surgeons to do a hemicolectomy at the time of diagnosis. I tried to reassure her that she had provided good care and such an operation would not have prevented what was happening now.”* (P1, FN)

Compassion was also seen in instances of patient advocacy beyond that expected: “*The nurse had finished shift, and knowing no friends/ family would be visiting, stayed with the patient longer.”* (P4, FN) These additional compassionate acts were sometimes discrete:

“*The fellow asked permission to lower the height of the bed so that we could sit down and be at eye level … at the end of the meeting, she asked if he would like the bed raised to the previous height.”* (P5, FN)

Other times, enacting compassion involved slowing down or deliberately connecting with a patient to an extent greater than needed to achieve technically adequate care:

“*The ICU fellow kneeled by her side and spoke in a gentle voice and told her it was ok, we were trying to help her, and could she please move onto the stretcher; she held out her hand and Mrs. C looked at her with a sense of trust and allowed her to help, and once on the stretcher would not let go of her hand.”* (P1, FN)

Adapting clinical care to integrate cultural beliefs and practices held by patients and their families was another way in which compassion was demonstrated as illustrated below for a patient of First Nations heritage.

*Her family arrived and gathered in the room. There was over 15 people… Her mother indicated she was the designated substitute decision-maker but she wanted to hear from all other family members about their perspective… It took a while to hear from everyone, some said only a few words, others spoke of her life and that she was returning to her ancestors… The family was first going to hold a sweet grass ceremony in the room and then would tell us when they were ready to discontinue life support; the nurse called security to disable the smoke alarms.* (P1, FN)

Changes in clinician *disposition* typically involved reprioritizing humanistic aspects of practice over the common technical focus. For instance, clinicians became more attuned and responsive to the emotions of patients and families, learners and colleagues, changed their tone of voice, used touch, or altered their posture to convey compassion. Other self-modifications involved being more present and reallocating time:

*“The medical student looked sad, and we talked for a while about patients dying and the impact on us, how it was sad that his family would not be with him; I told her she could be there with him if she wanted to. She decided that she wanted to sit with him, so that he would not die alone. While I finished the consult, she went back to the ICU, and she and the nurse sat by his bedside, even after he took his last breath.”* (P1, FN)

*Relational* modifications that conveyed compassion included spending additional time with a patient or communicating with them more frequently about non-clinical matters, including their hopes and aspirations, and acknowledging their past lives. Other relational modifications involved clinicians showing vulnerability, apologizing for misunderstandings, acknowledging different perspectives, responding to patient or family experiences of loss and suffering with empathy, and trying to understand the situation through the eyes of patients, family members, learners, and colleagues.

## Discussion

Grounded in the experiences and reflections of clinicians working in palliative care and critical care settings, our findings show how compassion can be conceptualized, observed, and expressed in different ways; we also describe how compassion can become more explicit through modulations in personal, clinical and relational activities*.* We found that expressions of compassion in clinical settings were multidirectional; they could be expressed between any two or more people, including patients, their families, and members of the healthcare team. In addition, expressions of compassion were responsive to emerging needs and circumstances, including clinical, social, cultural, procedural, and institutional. Expressions of compassion were reflected both in the states of mind of those involved, and in their actions, and included both practical skills and the intuitive expression and application of those skills within a given interaction. Compassion was conveyed through a holistic and integrative approach to care and professional practice. Expressions of compassion required being present to others’ experiences, connectedness, honesty, and accepting of them. These multiple expressions of compassion were identified in both critical care and palliative care settings, and in patient-clinician, learner-preceptor, and between-clinician interactions – this suggests that compassion is not discipline- or relationship specific, but rather, emerges in multiple contexts between many different actors.

A key finding from this study was that compassion does not yield well to taxonomic definitions; exceptions and variations confound a single concrete definition. Reconceptualizing compassion within a pattern language [31, 32] provides a much better fit with the polysemic and emergent nature of compassion we have described in our data. Pattern languages are adaptable and generative, allowing for new combinations of the component patterns as well as for some elements to be absent while still reflecting a shared pattern. There are similarities between the foundational elements of compassion identified in our study and those described in previous research with patients, [1] and clinicians, [33] specifically related to presence, acceptance, honesty, integration, connectedness, skill, art and enactment. Others have also identified personal, relational and systemic factors that can facilitate or introduce challenges to compassion, [34, 35] related to motivations, innate virtues, self-care, personal experiences of suffering, recipient responses, time, and competing demands. The contextual, emergent, and multidirectional aspects of compassion identified in our study are unique contributions to this discourse.

Participants in our study noted an undercurrent of compassion that was not necessarily appreciated in the moment and which only became apparent through subsequent reflection. This was most noticeable when acts of compassion extended beyond ‘routine’ care and engagement; clinicians adapted ‘normal’ processes of clinical care, perspectives and expectations, and ways of engaging with and responding to others in response to specific and often subtle dimensions of human experience. The capacity to notice when compassion is waning and adapt in the moment to restore compassion resonates with ecological theories of change [36]. An awareness of the undercurrent of compassion may help clinicians adapt to erosions of compassion by modulating themselves and the relational elements and processes of clinical care. How clinicians develop this capacity, how they determine which of these aspects to modulate to more explicitly express compassion, and how they modulate these aspects warrants further study.

Limitations include the study being situated within a single city within Western Canada and its sole focus on clinician perspectives. With compassion as a subjective experience, future research might explore patients’ and families’ perspectives, and how perceptions of compassion differ across cultures. To that end, the pattern language we have presented may not be complete or its facets stable across other contexts. This study is therefore a step along the way to establishing a more widely grounded pattern language of compassion in healthcare. We also acknowledge the self-report bias intrinsic to autoethnographic inquiry. However, we make no claim as to whether the perceptions and observations of our participants are objective or shared; it is the perceptual nature of compassion that frames our experience and understanding of it rather than its concrete expression. We also note that the palliative care field notes were on average twice the length of those from the ICU context and we had slightly more material from palliative care, which may have biased our interpretations to be more inclusive of palliative perspectives. We also note the large differences in the extent to which different participants contributed to the study, with more than half contributed by one participant. While this reflected the extent to which different participants were able to contribute, it also suggests more work is needed to test and validate the pattern language we have set out.

## Conclusions

Compassion is a complex and multifaceted phenomenon that is realized and perceived in many different ways. Our study reflects compassion as shifting patterns of attitudes, behaviours, and relationships grounded in highly situated circumstances, rather than as a single definable phenomenon. Compassion is expected within healthcare yet may be recognized when expressions of care are beyond ‘routine’. A pattern understanding of compassion as a phenomenon can help in shifting beyond single definitions.

## Additional files

Additional file 1 **Appendix 1.** Field notes template. (DOCX 15 kb) (DOCX 17 kb)

Additional file 2 **Appendix 2.** Interview guide. (DOCX 17 kb) (DOCX 15 kb)

## Abbreviations

FG: Focus group
FN: Field notes
ICU: Intensive care unit
IN: Interview

## References

[1] Sinclair S, McClement S, Raffin-Bouchal S, Hack TF, Hagen NA, McConnell S, Chochinov HM (2016). Compassion in health care: an empirical model. J Pain Symptom Manag.

[2] Chochinov HM (2007). Dignity and the essence of medicine: the a, B, C, and D of dignity conserving care. BMJ.

[3] Gilbert P (2009). The compassionate mind.

[4] Kay J (1990). Traumatic deidealization and the future of medicine. JAMA.

[5] Sinclair S, Norris JM, McConnell SJ, Chochinov HM, Hack TF, Hagen NA, McClement S, Bouchal SR (2016). Compassion: a scoping review of the healthcare literature. BMC Palliat Care.

[6] Irwin KE, Greer JA, Khatib J, Temel JS, Pirl WF (2013). Early palliative care and metastatic non-small cell lung cancer: potential mechanisms of prolonged survival. Chron Respir Dis.

[7] Kelley JM, Kraft-Todd G, Schapira L, Kossowsky J, Riess H (2014). The influence of the patient-clinician relationship on healthcare outcomes: a systematic review and meta-analysis of randomized controlled trials. PLoS One.

[8] Mannion R (2014). Enabling compassionate healthcare: perils, prospects and perspectives. Int J Health Policy Manag.

[9] Fotaki M (2015). Why and how is compassion necessary to provide good quality healthcare?. Int J Health Policy Manag..

[10] Cook D, Swinton M, Toledo F, Clarke F, Rose T, Hand-Breckenridge T, Boyle A, Woods A, Zytaruk N, Heels-Ansdell D, Sheppard R (2015). Personalizing death in the intensive care unit: the 3 wishes project: a mixed-methods study. Ann Intern Med.

[11] Epstein RM, Back AL (2015). A piece of my mind. Responding to suffering. JAMA.

[12] Joshi N. Doctor, shut up and listen. New York Times January 4th, 2015. pp. A17.

[13] CBC Radio. After near-death experience, doctor calls for more empathy with patients. 2017. http://www.cbc.ca/radio/asithappens/as-it-happens-thursday-edition-1.3922729/after-near-death-experience%2D%2D%20doctor-calls-for-greater-empathy-with-patients-1.3922731. Accessed 31 Jan 2019.

[14] Costa P, Magalhães E, Costa MJ (2013). A latent growth model suggests that empathy of medical students does not decline over time. Adv Health Sci Educ Theory Pract.

[15] Lown BA, Rosen J, Marttila J (2011). An agenda for improving compassionate care: a survey shows about half of patients say such care is missing. Health Aff (Millwood).

[16] Wigert H, Dellenmark Blom M, Bry K (2014). Parents’ experiences of communication with neonatal intensive-care unit staff: an interview study. BMC Pediatr.

[17] Wong P, Liamputtong P, Koch S, Rawson H (2015). Families’ experiences of their interactions with staff in an Australian intensive care unit (ICU): a qualitative study. Int Crit Care Nurs.

[18] Jones J, Winch S, Strube P, Mitchell M, Henderson A (2016). Delivering compassionate care in intensive care units: nurses’ perceptions of enablers and barriers. J Adv Nurs.

[19] Crawford P, Gilbert P, Gilbert J, Gale C, Harvey K (2013). The language of compassion in acute mental health care. Qual Health Res.

[20] Singleton V, Mee S (2017). Critical compassion: affect, discretion and policy-care relations. Sociol Rev.

[21] Patton MQ (1980). Qualitative evaluation methods.

[22] Charmaz K (2006). Constructing grounded theory: a practical guide through qualitative analysis.

[23] Bowen GA (2006). Grounded theory and sensitizing concepts. Int J Qual Methods.

[24] Engeström Y, Mietinnen R, Punamäki RL. Perspectives on activity theory. New York, NY: Cambridge University Press. p. 1999.

[25] Pawson R (2006). Evidence-based policy. A realist perspective.

[26] van Manen M (2007). Phenomenology of practice. Phenomenology & Practice.

[27] Hayano D (1979). Auto-ethnography: paradigms, problems and prospects. Hum Organ.

[28] Apramian T, Cristancho S, Watling C, Lingard L (2017). (Re)grounding grounded theory: a close reading of theory in four schools. Qual Res.

[29] Miles MB, Huberman M (2013). Qualitative Data Analysis.

[30] Bates J, Ellaway RH. Mapping the dark matter of context: a conceptual scoping review. Med Educ 2016;50:807–16Alexander C The Timeless Way of Building New York, NY: Oxford University Press; 1979. 10.1111/medu.13034 27402041

[31] Alexander C (1979). The timeless way of building.

[32] Ellaway RH, Bates J (2015). Exploring patterns and pattern languages of medical education. Med Educ.

[33] Sinclair S, Hack TF, Raffin-Bouchal S, McClement S, Stajduhar K, Singh P, Hagen NA, Sinnarajah A, Chochinov HM (2018). What are healthcare providers’ understandings and experiences of compassion? The healthcare compassion model: a grounded theory study of healthcare providers in Canada. BMJ Open.

[34] Kneafsey R, Brown S, Sein K, Chamley C, Parsons J (2015). A qualitative study of key stakeholders’ perspectives on compassion in healthcare and the development of a framework for compassionate interpersonal relations. J Clin Nurs.

[35] Singh P, Raffin-Bouchal S, McClement S, Hack TF, Stajduhar KA, Hagen NA, Sinnarajah A, Chochinov HM, Sinclair S. Healthcare providers’ perspectives on perceived barriers and facilitators of compassion: results from a grounded theory study. J Clin Nurs. 2018. 10.1111/jocn.14357 [Epub ahead of print]. 10.1111/jocn.14357 29575539

[36] Ellaway RH, Bates J, Teunissen PW (2017). Ecological theories of systems and contextual change in medical education. Med Educ.

