# Supplementary material for: A pattern language of compassion in intensive care and palliative care contexts
Source: BMC Palliat Care. 2019 Feb 2;18:15. doi: 10.1186/s12904-019-0402-0 (PMC6359837; doi:10.1186/s12904-019-0402-0)
Supplement: Supplementary file 1 — Appendix 1. Field notes template. (DOCX 15 kb) (DOCX 17 kb) [file 12904_2019_402_MOESM1_ESM.docx]

**Additional file 1: Appendix 1 – Field notes guide**

1. What was the organizational context for the interaction?
2. Who was involved in the observed interaction? What were their characteristics?
3. What did the different participants do?
4. Expressions of suffering
   1. Was suffering apparent and, if so, then who was suffering and how was their suffering expressed?
   2. What, if anything, facilitated the expression of suffering? (eg: people, objects, rules, routines)
   3. What, if anything, hindered the expression of suffering? (eg: people, objects, rules, routines)
   4. In what ways was suffering acknowledged?
   5. What responses were there to apparent suffering?
   6. In what ways was suffering not responded to?
5. Expressions of compassion
   1. In what ways was compassion expressed in this interaction?
   2. What, if anything, facilitated the expression of compassion? (eg: people, objects, rules, routines)
   3. What, if anything, hindered the expression of compassion? (eg: people, objects, rules, routines)
   4. What was the apparent impact of these expressions of compassion?
   5. Who was impacted by these expressions of compassion?
6. How did observing this interaction impact you?
7. Additional reflections and notes:
