# Supplementary material for: A pattern language of compassion in intensive care and palliative care contexts
Source: BMC Palliat Care. 2019 Feb 2;18:15. doi: 10.1186/s12904-019-0402-0 (PMC6359837; doi:10.1186/s12904-019-0402-0)
Supplement: Supplementary file 2 — Appendix 2. Interview guide. (DOCX 17 kb) (DOCX 15 kb) [file 12904_2019_402_MOESM2_ESM.docx]

**Additional file 2: Appendix 2 – Interview guide**

Thinking back to what you observed, what you thought, and what you wrote …

- What has been your experience of compassion in these contexts?
  - Do you perceive problems, challenges, and/or gaps in compassion in the contexts you have observed?
  - Did deliberately observing the presence or absence of compassion change your perspective on what compassion is and how you embody it?
- How does the enactment or performance of compassion change when moving from an ICU to a palliative care context?
  - What is it about the intensive care unit (ICU) context that enables, limits, shapes or changes the nature of compassion enacted there?
  - What is it about palliative care settings that enable, limit, shape or change the nature of compassion enacted there?
- Are there limits to compassion, especially in an interprofessional context when other professionals attend to different aspects of compassion?
  - Are there limits in compassion related to scope of practice?
  - Who is responsible for compassion? For example, do you need to enact compassion or do you need to ensure that others experience compassion but not necessarily from you?
- After participating in this study, what is your view on compassion as active (transformative, assertive) or passive (given, ambient, present)?
